# Erectile dysfunction, physical activity and metabolic syndrome: differences in markers of atherosclerosis

**DOI:** 10.1186/1471-2261-11-36

**Published:** 2011-06-27

**Authors:** Hanna Pohjantähti-Maaroos, Ari Palomäki, Juha Hartikainen

**Affiliations:** 1 Department of Emergency Medicine, Kanta-Häme Central Hospital, Ahvenistontie 20, FI-13530, Hämeenlinna, Finland; 2 Department of Cardiometabolic Research, Linnan Klinikka, Raatihuoneenkatu 10, FI-13100, Hämeenlinna, Finland; 3 Heart Center, Kuopio University Hospital, PL 1777, FI-70211 Kuopio, Finland

## Abstract

**Background:**

Erectile dysfunction (ED), impaired arterial elasticity, elevated resting heart rate as well as increased levels of oxidized LDL and fibrinogen associate with future cardiovascular events. Physical activity is crucial in the prevention of cardiovascular diseases (CVD), while metabolic syndrome (MetS) comprises an increased risk for CVD events. The aim of this study was to assess whether markers of subclinical atherosclerosis are associated with the presence of ED and MetS, and whether physical activity is protective of ED.

**Methods:**

57 MetS (51.3 ± 8.0 years) and 48 physically active (PhA) (51.1 ± 8.1 years) subjects participated in the study. ED was assessed by the International Index of Erectile Function (IIEF) questionnaire, arterial elasticity by a radial artery tonometer (HDI/PulseWave™ CR-2000) and circulating oxLDL by a capture ELISA immunoassay. Fibrinogen and lipids were assessed by validated methods. The calculation of mean daily energy expenditure of physical exercise was based on a structured questionnaire.

**Results:**

ED was more often present among MetS compared to PhA subjects, 63.2% and 27.1%, respectively (p < 0.001). Regular physical exercise at the level of > 400 kcal/day was protective of ED (OR 0.12, 95% CI 0.017-0.778, p = 0.027), whereas increased fibrinogen (OR 4.67, 95% CI 1.171-18.627, p = 0.029) and elevated resting heart rate (OR 1.07, 95% CI 1.003-1.138, p = 0.04) were independently associated with the presence of ED. In addition, large arterial elasticity (ml/mmHgx10) was lower among MetS compared to PhA subjects (16.6 ± 4.0 *vs*. 19.6 ± 4.2, p < 0.001), as well as among ED compared to non-ED subjects (16.7 ± 4.6 *vs*. 19.0 ± 3.9, p = 0.008). Fibrinogen and resting heart rate were highest and large arterial elasticity lowest among subjects with both MetS and ED.

**Conclusions:**

Markers of subclinical atherosclerosis associated with the presence of ED and were most evident among subjects with both MetS and ED. Thus, especially MetS patients presenting with ED should be considered at high risk for CVD events. Physical activity, on its part, seems to be protective of ED.

**Trial registration:**

ClinicalTrials.gov NCT01119404

## Background

Atherosclerosis begins with oxidation of LDL particles in the arterial wall [1]. Oxidatively modified LDL (oxLDL) damages the endothelium of the artery - a pathophysiology similar to that of vascular erectile dysfunction (ED) [1,2]. As a result, the elasticity of the arteries deteriorates. Impaired arterial elasticity and increased levels of circulating oxLDL as well as elevated fibrinogen and resting heart rate associate with subclinical atherosclerosis and increased risk of cardiovascular disease (CVD) events [3-8].

Besides similar pathophysiology, ED and CVD share same risk factors [9]. In addition, a high prevalence of both silent and clinical CVD has been reported among ED patients [9,10]. ED has also been reported as an independent predictor of incident CVD [11,12]. Since ED often precedes CVD symptoms from other vascular beds, it is thought to be an early clinical manifestation of systemic atherosclerosis [9,13].

Physical activity is known to be crucial in the prevention of CVD. Sedentary lifestyle, on its part, predisposes to metabolic syndrome (MetS), a clustering of metabolic disorders; visceral obesity, hypertension, dyslipidaemia and insulin resistance or diabetes [14]. MetS comprises a high risk for CVD events even in the absence of diabetes [15]. Mechanisms that link MetS to increased CVD risk are, however, incompletely understood.

In the present study we assessed arterial elasticity, circulating oxLDL levels, fibrinogen and resting heart rate among MetS and physically active (PhA) subjects. The aim was to study whether these markers of subclinical atherosclerosis associate with ED and MetS, and whether physical activity is protective of ED.

## Methods

### Subjects

120 men with MetS and 80 physically active (PhA) men participating in the Hämeenlinna Metabolic Syndrome research program (HMS) were recruited in the study. MetS was diagnosed according to National Cholesterol Education Program (NCEP) criteria [16]. We interviewed the subjects on their medical history and lifestyle habits. Participation of a PhA subject was accepted if he exercised more than three times a week and 30 minutes per exercise on a regular basis without chest pain, dyspnea or fatigue, and did not fulfil the criteria of MetS. Exclusion criteria were non-specific beta-blocker medication and suspected non-vascular ED. Suspicion of non-vascular ED was based on patient records and patients' self-report during in the presence of possible psychogenic, urogenital, neurological or endocrinological cause for ED. Diagnoses of diabetes, hypertension and CVD were based on patients' report on previously diagnosed diseases, patient records and the use of antihyperglycemic, antihypertensive or antianginal medication. Positive family history of CVD was considered among subjects reporting previously diagnosed CVD in a first degree relative.

Subjects filled in a structured questionnaire on their average amount, type and intensity of leisure time physical exercise per week. The compendium of physical activities and subjects' self-rated intensity levels were used in estimating the metabolic equivalent (MET) values [17]. The energy expenditure of mean daily physical exercise was calculated in kilocalories by multiplying the MET value and exercise times per week and mean duration of exercise in hours and person's weight in kilograms and finally dividing it by seven. Physical activity level was considered low if mean daily energy expenditure of physical exercise was < 200 kcal/day, moderate if 200-400 kcal/day and high if > 400 kcal/day. In addition, waist circumference, height, weight and blood pressure were measured.

Each study subject signed an informed consent. The ethics committee of the Kanta-Häme Hospital District in Finland approved the study which was carried out in compliance with the Helsinki Declaration.

### International Index of Erectile Dysfunction (IIEF) questionnaire

Subjects filled in the IIEF questionnaire [18]. The sum of the questions 1-5 and 15 was calculated to assess the presence of ED. Subjects with maximal score of 30 were considered to have normal erectile function and subjects with score of ≤25, were considered to have ED. To ensure that the study subjects truly had either completely normal or impaired erectile function, subjects with IIEF score 26 to 29 were excluded. The question number 15 (how do you rate your confidence that you can get and keep your erection?) was used to assess the presence of erectile function in men reporting lack of sexual activity in questions 1-5. A subject reporting very high confidence in the question number 15, was considered to have normal erectile function. Those reporting very low to medium confidence were considered to have ED, and those reporting high confidence were excluded.

### Arterial elasticity and laboratory procedures

Arterial elasticity was assessed by a non-invasive radial artery tonometer (HDI/PulseWave™CR-2000) in a semi-sitting position. Arterial tonometer uses a modified Windkessel method to estimate systemic large (C1) and small (C2) arterial elasticity [3]. C1 identifies the elasticity of the aorta and other large arteries, C2 the elasticity and endothelial function of the microvascular circulation. Indices determined by this validated method correlate tightly with those determined invasively [3,19]. In addition, C1 seems to correlate significantly with MRI-determined aortic distensibility, whereas C2 correlate with endothelial function assessed by flow-mediated dilation [20,21]. In addition, elasticity indices assessed by the HDI/Pulsewave™CR-2000 have been reported reliable and repeatable over a short and long period of observation [19,22]. The reference values depend on age and gender. Among men aged 50-59 years, C1 above 11 ml/mmHgx10 and C2 above 7 ml/mmHgx100 are considered normal.

Arterial elasticity indices were assessed automatically by the tonometer as a mean of five most similar pulse waves appearing during 30 seconds of measurement. Mean of four consecutive measurements was assessed. Blood pressure and resting heart rate were automatically measured by the CR-2000 during the elasticity measurement. Intraindividual CV% was 9.0% for C1 and 8.8% for C2. Same experienced nurse performed all measurements.

Plasma levels of oxLDL were determined as duplicates by a capture ELISA immunoassay (Mercodia AB, Uppsala, Sweden). It uses the same monoclonal antibody as in the assays by Holvoet et al [6]. CV% of oxLDL measurement was 7.7%. Fibrinogen and lipids were assessed by validated methods.

### Statistical methods

Statistics were analyzed with SPSS for Windows 17.0. Data are presented as mean ± SD if not mentioned otherwise. A probability value < 0.05 was considered statistically significant.

Student's T-test was used in assessing the differences between subjects with ED and normal erectile function as well as between MetS and PhA subjects in case of normality. Mann Whitney U-test was used in case of non-normality. ANOVA was used to analyze the adjusted p values for differences in two-dimensional variables as well as in four-dimensional variables in case of normality. Bonferroni post hoc analysis was used for multiple comparisons regarding fibrinogen, resting heart rate and arterial elasticity. The differences were adjusted for age, smoking, blood pressure, diabetes, CVD, and LDL cholesterol. Kruskall-Wallis test was used in case of non-normality. Differences in categorical values were calculated by χ^2 ^test. Univariate and multivariate analyses of the associations between risk factors and markers of CVD and the presence of ED were conducted with binary logistic regression model. Traditional CVD risk factors and assessed markers of subclinical atherosclerosis without strong correlation with each other were included as covariates in the multivariate analysis. Multivariate analysis was conducted with the enter method by removing covariates without association to ED one by one. The result was verified with the forward conditional method and adjusted for the use of medications. OR, 95% CI and p values for covariates in the univariate analyses and for significant covariates in the adjusted multivariate analysis, as well as Nagelkerke R^2 ^of the final model are presented.

## Results

**Figure 1:**
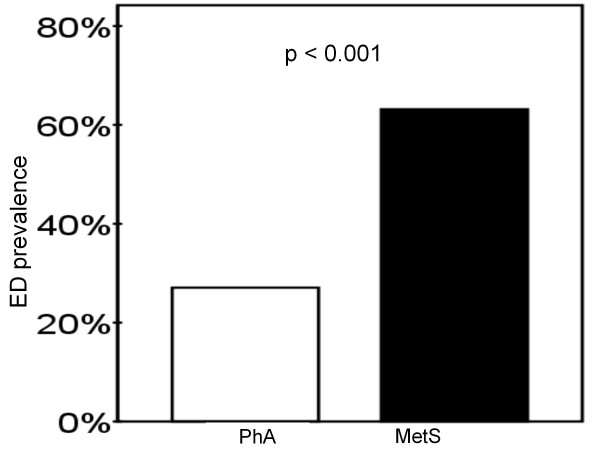
**Prevalence of erectile dysfunction (ED) among metabolic syndrome (MetS) and physically active (PhA) subjects**.

Fifty-seven MetS and 48 PhA subjects without exclusion criteria completed the IIEF questionnaire. Ten men with MetS and four PhA men reported lack of sexual activity. All except one of these 14 men were diagnosed with ED according to the question number 15. Altogether ten men with MetS had previously diagnosed CVD. There were four patients with coronary artery disease, three with cerebrovascular disease, two with coronary artery and cerebrovascular disease and one with coronary artery and peripheral artery disease. ED was more often present among MetS compared to PhA subjects, 36 (63.2%) and 13 (27.1%), respectively (p < 0.001) (Figure 1). Clinical characteristics and medications of the study groups are presented in Table 1 and clinical chemistry in Table 2. None of the study subjects used PDE5-inhibitors.

**Table 1:** Clinical characteristics of study subjects.


	**PhA**		**MetS**		
	**no ED**	**ED**	**no ED**	**ED**	**p**
	**(n = 35)**	**(n = 13)**	**(n = 21)**	**(n = 36)**	

Age^1^, years	49.4 ± 7.5	56.9 ± 6.7	45.9 ± 4.6	54.1 ± 8.2	< 0.001
CVD in family, n (%)	18 (51.4%)	9 (69.0%)	1 (52.3%)	18 (50.0%)	NS
Diagnosed CVD^b^, n (%)	0 (0%)	0 (0%)	3 (14.3%)	7 (19.4%)	< 0.05
Diabetics^2,a^, n (%)	0 (0%)	0 (0%)	7 (33.3%)	19 (52.8%)	< 0.001
Hypertension^2,a^, n (%)	1 (2.9%)	3 (23.1%)	11 (52.4%)	20 (55.6%)	< 0.001
ASA^c^, n (%)	4 (11.4%)	0 (0%)	5 (23.8%)	10 (27.8%)	NS
β_1_-blocker^2,a^, n (%)	0 (0%)	1 (7.7%)	4 (19.0%)	15 (41.7%)	< 0.001
ACE-inhibitor, n (%)	1 (2.9%)	2 (15.4%)	2 (9.5%)	4 (11.1%)	NS
ATR-blocker^a^, n (%)	0 (0%)	0 (0%)	5 (23.8%)	11 (30.6%)	< 0.01
Ca-blocker, n (%)	0 (0%)	1 (7.7%)	1 (4.8%)	3 (8.3%)	NS
Diuretic^b^, n (%)	0 (0%)	1 (7.7%)	4 (19.0%)	8 (22.2%)	< 0.05
Statin^b^, n (%)	4 (11.4%)	2 (15.4%)	7 (33.3%)	14 (38.9%)	< 0.05
Smoking^b^					< 0.01
-current, n (%)	1 (2.9%)	0 (0%)	5 (23.8%)	6 (16.7%)	
-former, n (%)	11 (31.4%)	6 (46.2%)	10 (47.6%)	18 (50.0%)	
-never, n (%)	23 (65.7%)	7 (53.8%)	6 (28.6%)	12 (33.3%)	
Physical activity^2,a^, kcal/day	506.9 ± 300	467.1 ± 256	209.4 ± 265	156.4 ± 172	< 0.001
Alcohol intake^b^, g/day	7.8 ± 6.0	6.0 ± 4.3	15.0 ± 13.5	20.1 ± 22.8	< 0.01
BMI^2,a^, kg/m^2^	23.8 ± 2.0	24.6 ± 3.0	32.7 ± 4.5	32.0 ± 4.8	< 0.001
Waist circumf.^2,a^, cm	88.6 ± 7.1	90.8 ± 8.9	113.5 ± 10.5	114.8 ± 13.3	< 0.001
SBP^3,a^, mmHg	127.2 ± 9.2	126.9 ± 11.2	136.5 ± 12.2	139.8 ± 16.5	< 0.001
DBP^a^, mmHg	73.9 ± 6.7	74.5 ± 5.6	82.1 ± 7.4	81.7 ± 8.8	< 0.001

^1-3 ^p value for the difference between men with and without ED: ^1 ^p < 0.001, ^2 ^p < 0.01, ^3 ^p < 0.05. NS if not mentioned.

^a-c ^p value for the difference between PhA and MetS subjects: ^a ^p < 0.001, ^b ^p < 0.01, ^c ^p < 0.05. NS if not mentioned.

ASA - acetylsalicylic acid; ATR - angiotensin receptor; BMI - body mass index; CVD - cardiovascular disease; DBP - diastolic blood pressure; ED - erectile dysfunction; MetS - metabolic syndrome; PhA - physically active; SBP - systolic blood pressure

**Table 2:** Clinical chemistry of study subjects.

	PhA		MetS		
	no ED	ED	no ED	ED	p
	(n = 35)	(n = 13)	(n = 21)	(n = 36)	
Cholesterol, mmol/L	5.27 ± 0.7	5.33 ± 0.8	5.26 ± 1.1	5.30 ± 1.6	NS
LDL-C, mmol/L	3.47 ± 0.7	3.32 ± 0.9	3.24 ± 0.9	3.21 ± 1.0	NS
HDL-C^3,a^, mmol/L	1.61 ± 0.3	1.81 ± 0.4	1.20 ± 0.2	1.16 ± 0.3	< 0.001
Triglycerides^3,a^, mmol/L	0.94 ± 0.5	0.86 ± 0.4	2.49 ± 1.4	3.19 ± 5.1	< 0.001
OxLDL, U/L	71.8 ± 21.4	75.5 ± 35.5	78.5 ± 40.1	80.8 ± 33.5	NS
Glucose^2,a^, mmol/L	5.49 ± 0.4	5.59 ± 0.6	6.54 ± 1.0	6.99 ± 2.2	< 0.001
HbA1C^2,a^,%	5.61 ± 0.2	5.60 ± 0.2	6.03 ± 0.6	6.47 ± 1.2	< 0.001

ED - erectile dysfunction; HbA1C - glycosylated haemoglobin; HDL-C - high density lipoprotein cholesterol; LDL-C - low density lipoprotein cholesterol; MetS - metabolic syndrome; OxLDL - oxidized LDL; PhA - physically active. Upper indices for p values as in Table 1.

There was an evident difference in the amount of daily physical exercise between PhA and MetS subjects, 496.7 ± 286.9 kcal/day *vs*. 176.3 ± 210.8 kcal/day, respectively (p < 0.001). In addition, subjects with normal erectile function were physically more active compared to subjects with ED, 395.3 ± 319.5 kcal/day *vs*. 235.7 ± 237.3 kcal/day, respectively (p = 0.005). Physical activity of the study groups is presented in Table 1.

**Figure 2:**
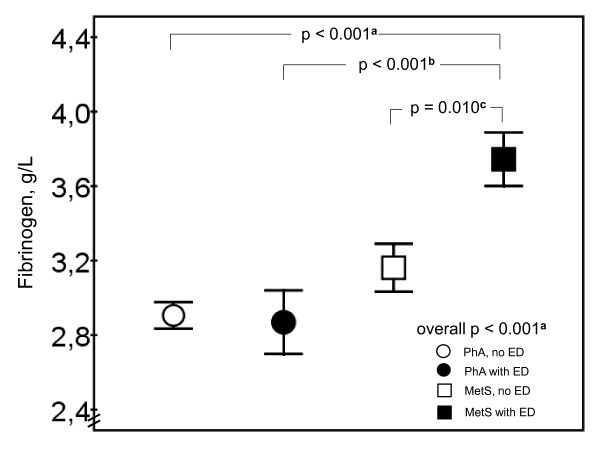
**Fibrinogen levels (g/L) between the study groups (n = 105)**. Mean ± SEM is presented. P values after adjustment for age, smoking, blood pressure, established CVD, diabetes and LDL cholesterol, ^a ^p < 0.001, ^b ^p < 0.01, ^c ^p < 0.05.

Fibrinogen was lower among PhA compared to MetS subjects, 2.90 ± 0.47 g/L *vs*. 3.53 ± 0.82 g/L, respectively (p < 0.001). Furthermore, fibrinogen levels were lower among subjects with normal erectile function compared to those with ED, 3.00 ± 0.50 g/L *vs*. 3.51 ± 0.89 g/L, respectively (p = 0.001). Fibrinogen levels were 2.91 ± 0.4 g/L among PhA subjects without ED, 2.87 ± 0.6 g/L among PhA subjects with ED, 3.16 ± 0.6 g/L among MetS subjects without ED, and 3.74 ± 0.9 g/L among MetS subjects with ED (Figure 2). There were no significant differences in oxLDL levels between any of the groups (Table 2).

**Figure 3:**
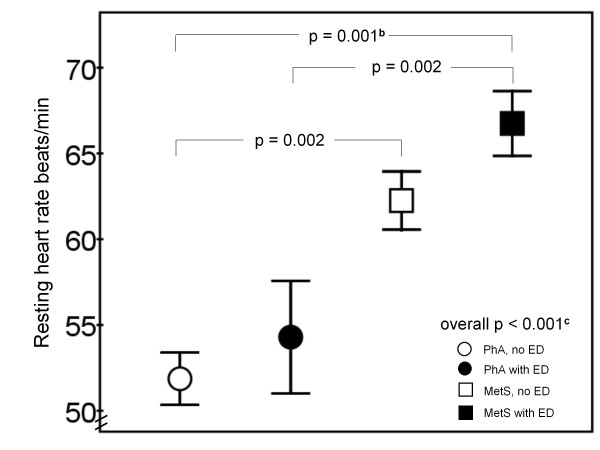
**Resting heart rate (beats/min) between the study groups (n = 105)**. Mean ± SEM is presented. P values after adjustment for age, smoking, blood pressure, established CVD, diabetes and LDL cholesterol, ^b ^p < 0.01, ^c ^p < 0.05.

Resting heart rate was lower among PhA compared to MetS subjects, 52.5 ± 9.6 beats/min *vs*. 65.1 ± 10.0 beats/min (p < 0.001). The difference remained significant after adjustment for age and selective β-blocker medication (p < 0.001). Resting heart rate was also lower among subjects with normal erectile function, compared to those with ED, 55.6 ± 9.8 beats/min *vs*. 63.5 ± 12.3 beats/min (p = 0.001). After adjustment for age and selective β-blocker medication, the difference remained significant (p = 0.001). Resting heart rate was 51.9 ± 9.0 beats/min among PhA subjects without ED, 54.3 ± 11.4 beats/min among PhA subjects with ED, 62.3 ± 7.6 beats/min among MetS subjects without ED, and 66.8 ± 11.0 beats/min among MetS subjects with ED (Figure 3).

**Figure 4:**
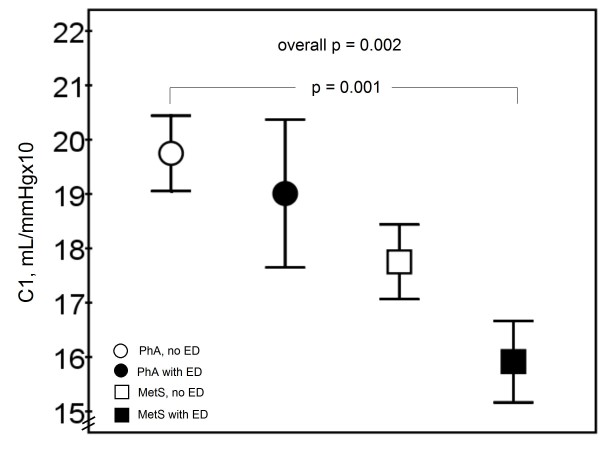
**Large arterial elasticity (C1, ml/mmHgx10) between the study groups (n = 105)**. Mean ± SEM is presented. After adjustment for age, smoking, blood pressure, established CVD, diabetes and LDL cholesterol, the differences were not significant.

PhA subjects had better large arterial elasticity (C1) compared to MetS subjects, 19.6 ± 4.2 ml/mmHgx10 and 16.6 ± 4.0 ml/mmHgx10, respectively (p < 0.001). There was no difference between the groups in small arterial elasticity (C2). Subjects with normal erectile function had better C1 compared to those with ED, 19.0 ± 3.9 ml/mmHgx10 and 16.7 ± 4.6 ml/mmHgx10, respectively (p = 0.008). Also C2 was better among those with normal erectile function compared to those with ED, 7.5 ± 3.2 ml/mmHgx100 and 6.2 ± 3.0 ml/mmHgx100, respectively (p = 0.035). The difference in C1 remained significant even after adjustment for age (p = 0.035) and the use of selective β-blockers (p = 0.026). After the same adjustment the difference in C2 did not remain significant. C1 was 19.7 ± 4.1 ml/mmHgx10 among PhA subjects without ED, 19.0 ± 4.7 ml/mmHgx10 among PhA subjects with ED, 17.8 ± 3.1 ml/mmHgx10 among MetS subjects without ED and 15.9 ± 4.4 ml/mmHgx10 among MetS subjects with ED (Figure 4). C2 was 7.2 ± 2.7 ml/mmHgx100, 6.7 ± 3.3 ml/mmHgx100, 8.1 ± 3.9 ml/mmHgx100, and 6.0 ± 2.9 ml/mmHgx100, respectively (NS).

Results of the univariate analyses are presented in Table 3. MetS associated with the presence of ED even after adjustment for age, physical activity, smoking and total cholesterol (OR = 5.83, 95% CI 1.730-19.618, p = 0.004). The association of MetS and ED did not remain significant after adjustment for individual MetS components. Of the markers of subclinical atherosclerosis, C1 and C2 were negatively and fibrinogen and resting heart rate positively associated with the presence of ED. The association between ED and C1 remained significant after adjustment for age (p = 0.047). OxLDL levels did not associate with the presence of ED.

**Table 3:** Univariate analyses of risk factors and markers of CVD as predictors of ED.

Covariates	OR	95% CI	p
Age, years	1.14	1.066-1.209	< 0.001
Smoking, yes/no	1.16	0.349-3.871	NS
No of pack-years in smokers	1.06	1.005-1.116	0.031
Metabolic syndrome, yes/no	4.62	2.005-10.623	<0.001
Diabetes, yes/no	4.43	1.666-11.794	0.003
Hypertension, yes/no	3.24	1.387-7.587	0.007
CVD, yes/no	2.94	0.718-12.082	NS
Family history of CVD, yes/no	1.14	0.529-2.466	NS
BMI, kg/m^2^	1.11	1.029-1.200	0.007
Physical activity, >400 vs. <200 kcal/day	0.22	0.082-0.561	0.002
HDL cholesterol, mmol/L	0.47	0.177-1.239	NS
LDL cholesterol, mmol/L	0.82	0.524-1.293	NS
Triglycerides, mmol/L	1.31	0.948-1.802	NS
Heart rate, beats/min	1.07	1.026-1.110	0.001
Fibrinogen, g/L	2.83	1.517-5.274	0.001
C1, ml/mmHgx10	0.88	0.794-0.969	0.010
C2, ml/mmHgx100	0.87	0.756-0.993	0.040
OxLDL, U/L	1.00	0.991-1.016	NS

BMI - body mass index; C1 -large arterial elasticity; C2 - small arterial elasticity; CVD - cardiovascular disease; ED - erectile dysfunction; HDL -high density lipoprotein; LDL - low density lipoprotein; OxLDL - oxidized low density lipoprotein

In the multivariate analysis among all study subjects, age, fibrinogen and resting heart rate were directly and physical activity > 400 kcal/day inversely associated with the presence of ED. These covariates remained significant even after adjustment for traditional CVD risk factors and medications. Results of the adjusted multivariate analysis are presented in Table 4.

**Table 4:** Significant predictors of ED in the multivariate analysis.

Covariates	OR	95% CI	p
Age, years	1.19	1.072-1.322	0.001
Heart rate, beats/min	1.07	1.003-1.138	0.040
Physical activity, >400 vs. <200 kcal/day	0.12	0.017-0.778	0.027
Fibrinogen, g/L	4.67	1.171-18.627	0.029

The model was adjusted for smoking, CVD, diabetes, hypertension, family history of CVD, BMI, HDL and LDL cholesterol, triglycerides and medications. R^2 ^= 0.546.

In the multivariate analysis among MetS subjects only, age (OR 1.23, 95% CI 1.09-1.39, p = 0.001) and fibrinogen (OR 4.30 95% CI 1.21-15.2, p = 0.024) associated directly and physical activity > 400 kcal/day (OR 0.05, 95% CI 0.004-0.65, p = 0.022) inversely with the presence of ED. These covariates were significant predictors of ED even after adjustment for smoking, diabetes, hypertension, CVD, LDL and HDL cholesterol, triglycerides, BMI, family history of CVD, and selective beta-blockers. Among PhA subjects, age was the only significant predictor for the presence of ED (OR 1.16, 95% CI 1.04-1.30, p = 0.008).

## Discussion

In the present study, physical activity was an independent predictor of normal erectile function, whereas increased fibrinogen and resting heart rate associated independently with erectile dysfunction (ED). Impaired large arterial elasticity (C1) was related to the presence of ED as well as to the presence of MetS. Markers of subclinical atherosclerosis were most evident among subjects with both MetS and ED.

Previously physical activity has been found to decrease the risk of ED and to improve sexual function among those with established ED [23-25]. We found a physical exercise level of > 400 kcal/day (*i.e*. > 2800 kcal/week) to associate independently with normal erectile function in the analyses among all participants as well as in the analyses restricted to MetS subjects only. To our knowledge, this is the first study reporting the positive association between physical activity and the presence of normal erectile function among MetS.

PhA subjects presented often with normal erectile function, whereas ED was highly prevalent among men with MetS. Although we excluded subjects with submaximal IIEF score (score 26-29), still considered as normal erectile function by the IIEF, the presence of ED among PhA subjects was lower than expected [26]. Since obesity, smoking and excess alcohol consumption have been reported to associate with ED, and physical activity is protective of ED, the overall healthy behaviour of the PhA subjects must have contributed to the high existence of normal erectile function among them [23-25,27].

In a recent study, MetS did not improve prediction of CVD after adjustment for its individual components [28]. In agreement, MetS *per se *was not an independent predictor of ED in the present study. However, we found a high prevalence of ED among MetS which has also been reported previously [29]. The prevalence of ED in the presence of diabetes or CVD is known to be even higher [13,30]. Accordingly, there were more diabetics and CVD patients among those with MetS and ED in the present study. Those with ED were also older and more often hypertensive. Although subjects with non-selective β-blocker medication were excluded, ED subjects were more often on selective beta-blockers. These factors may contribute to the higher number of ED among MetS, and thereby to the findings of the study. However, neither diabetes nor CVD were significant predictors of ED in the multivariate analyses of the present study. In addition, although the risk of sexual dysfunction caused by β-blockers was low in a previous systematic review of randomized trials, the results of the present study were adjusted for the use of selective beta-blockers [31]. Furthermore, also medications reported to improve sexual function were more often used among subjects with MetS and ED [32,33]. Thus, the high use of medications among MetS seems rather reflect the physical inactivity, obesity and concomitant diseases among them, whereas the high prevalence of ED among MetS may reflect the presence of underlying, atherosclerotic disease.

We found increased fibrinogen levels among MetS compared to PhA subjects which agrees with previous studies [34,35]. Physical inactivity, obesity and smoking have been reported to associate with increased fibrinogen levels, and thus may partly explain the difference [35-37]. Fibrinogen levels were significantly higher also in the presence of ED compared to the levels in the presence of normal erectile function in the analyses among all participants but also among only MetS subjects. In addition, increased fibrinogen levels associated with the presence of ED independently of multiple other CVD risk factors. There are no previous studies on the association of increased fibrinogen and ED among MetS. However, our finding agrees with a previous study by Vlachopoulos et al [38] reporting an independent predictive value of fibrinogen for the presence of ED both among men with and without coronary artery disease. Differing from the analyses restricted to MetS subjects only, fibrinogen levels did not associate with the presence of ED among PhA subjects in the present study. The reason may be the small number of ED among them, again reflecting the protective effect of healthy, active lifestyle.

In line with previous publications we found a decrease in large arterial elasticity (C1) among MetS compared to PhA subjects [39,40]. In addition, impaired C1 associated with the presence of ED. C1 was especially low among men with both MetS and ED. There are also previous reports on the connection between impaired large arterial elasticity and ED [41,42]. However, in these studies arterial elasticity was assessed by ultrasound or pulse-wave velocity measurements, not by the pulse-wave analysis as in our previous and the present study [43].

Endothelial dysfunction is believed to be a key mechanism in the pathogenesis of ED as well as of other atherosclerotic cardiovascular diseases [1,2]. In studies assessing endothelial function by a regional measurement of brachial flow-mediated dilation, endothelial dysfunction associated with ED [44,45]. Small arterial elasticity (C2), assessed in the present study, reflects the systemic endothelial function of the microvascular circulation but is also affected by alterations in the elastic properties of the arterial wall [3]. Since C2 is not a mere measurement of endothelial function, it may explain why the association between C2 and the presence of ED did not remain significant after adjustments.

Regular aerobic exercise may attenuate age-related reduction in large arterial elasticity [46] and decrease circulating oxLDL levels [47]. However, a significant decrease in arterial elasticity and increase in oxidative stress have been reported after exercise at a vigorous level [48,49]. In our study, large arterial elasticity was better among PhA compared to MetS subjects whereas oxLDL levels were comparable between the groups. The relatively small number of study subjects may explain why the trend of increasing oxLDL in the presence of MetS and ED remained non-significant. In addition, although the inclusion criterion of minimum physical exercise for the PhA subjects was relatively low, the reported high level and intensity of exercise among them might have influenced the results.

Previously MetS variables, such as hypertension, insulin resistance and obesity, have been reported to associate with adrenergic overdrive [50]. Accompanying elevation in resting heart rate increases peak blood flow during diastole which in turn enhances pulsatile and shear stress on the endothelium [51]. Resulting endothelial dysfunction and loss of arterial elasticity may explain the connection between elevated resting heart rate and ED [51,52]. In agreement, resting heart rate was higher among MetS compared to PhA subjects in the present study, despite the higher use of beta-blockers among MetS. In addition, heart rate was higher among ED compared to non-ED subjects, being highest among men with both MetS and ED. Since MetS subjects with ED were physically most inactive, the elevated resting heart rate was at least partly a consequence of their physical unfitness. However, elevated resting heart rate predicted the presence of ED independently of physical activity, multiple other CVD risk factors, and beta-blocker medication.

Because of the cross-sectional design, we cannot make assumptions on the linkage between the markers of subclinical CVD and true CVD risk in the future. However, the present study provides possible pathophysiological links between erectile dysfunction and increased cardiovascular risk among MetS. Subjects with MetS are known to be at high risk for CVD [15]. ED, on its part, is considered as an early clinical manifestation of systemic atherosclerosis [9,13]. Markers of subclinical atherosclerosis, assessed in the present study, have previously been reported to associate with increased risk of CVD events and mortality [4,5,7,8]. Since increased fibrinogen and elevated resting heart rate as well as impaired arterial elasticity were present among MetS compared to PhA subjects, as well as among ED compared to non-ED subjects, they may partly explain the increased CVD risk associated both with MetS and with ED. Since these markers of subclinical atherosclerosis were most evident among subjects with both MetS and ED, especially these patients should be considered at high cardiovascular risk.

One mechanism linking MetS to ED is believed to be hypogonadism associated with visceral obesity [53]. In addition, physical activity may partly intermediate its benefits through an induced increase in testosterone levels [54]. Another possible pathophysiologic linkage between MetS and ED is subclinical inflammation [55]. We did not assess testosterone or CRP levels which was a limitation of the study.

IIEF questionnaire is a reliable method to assess the presence and severity of ED [56]. In our study, number of subjects reported lack of sexual activity and thus were unable to answer the IIEF questions concerning erectile function during intercourse. Among them, we used a single-question assessment of ED. Although the single-question assessment has been found to properly identify men with ED [57], we wanted to be sure that subjects participating in the study truly had either completely normal erectile function or real ED. Therefore, we accepted only men with either full score or score ≤25 in the six-question IIEF or men with full score or score ≤3 in the single-question assessment to participate in the study. Because of the relatively small number of study subjects and the fact that a single-question assessment of ED was used among subjects without previous sexual activity, we could not study the effects of severity of ED.

## Conclusions

Increased fibrinogen and resting heart rate as well as impaired arterial elasticity associated with the presence of ED and were most evident among subjects with both MetS and ED. Physical exercise, on its part, was a strong and independent predictor of normal erectile function among all as well as among only MetS subjects. Thus, especially MetS patients presenting with ED should be considered at high risk for CVD. In addition, our findings support the importance of physical exercise in the management of MetS and concomitant diseases.

## Competing interests

The authors declare that they have no competing interests.

## Authors' contributions

AP designed the study. HPM and AP participated in the acquisition and analysis of the data. HPM, AP and JH participated in the drafting of the manuscript. All authors have approved the final manuscript.

## Pre-publication history

The pre-publication history for this paper can be accessed here:

http://www.biomedcentral.com/1471-2261/11/36/prepub

## References

[B1] Stocker R Keaney JF Jr Role of oxidative modifications in atherosclerosis Physiol Rev 2004 84 1381 1478 10.1152/physrev.00047.2003 15383655

[B2] Kirby M Jackson G Simonsen U Endothelial dysfunction links erectile dysfunction to heart Disease Int J Clin Pract 2005 59 225 229 10.1111/j.1742-1241.2005.00453.x 15854201

[B3] Cohn J Finkelstein S McVeigh G Morgan D LeMay L Robinson J Mock J Non-invasive Pulse Wave Analysis for the Early Detection of Vascular Disease Hypertension 1995 26 503 508 7649589 10.1161/01.hyp.26.3.503

[B4] Boutouyrie P Tropeano I Asmar R Gautier I Benetos A Lacolley P Laurent S Aortic stiffness is an independent predictor of primary coronary events in hypertensive patients. A longitudinal study Hypertension 2002 39 10 15 10.1161/hy0102.099031 11799071

[B5] Van Popele N Grobbee D Bots M Asmar R Topouchian J Reneman R Hoeks A Van der Kuip D Hofman A Witteman J Association between arterial stiffness and atherosclerosis. The Rotterdam study Stroke 2001 32 454 460 10.1161/01.STR.32.2.454 11157182

[B6] Holvoet P Mertens A Verhamme P Bogaerts K Beyens G Verhaeghe R Collen D Muls E Van de Werf F Circulating oxidized LDL is a useful marker for identifying patients with coronary artery disease Arterioscler Thromb Vasc Biol 2001 21 844 848 10.1161/01.ATV.21.5.844 11348884

[B7] Fibrinogen Studies Collaboration Plasma fibrinogen level and the risk of major cardiovascular diseases and nonvascular mortality: an individual participant meta-analysis JAMA 2005 294 1799 1809 16219884 10.1001/jama.294.14.1799

[B8] Cooney M Vartiainen E Laatikainen T Juolevi A Dudina A Graham I Elevated resting heart rate is an independent risk factor for cardiovascular disease in healthy men and women Am Heart J 2010 159 612 619 10.1016/j.ahj.2009.12.029 20362720

[B9] Jackson G Boon N Eardley I Kirby M Dean J Hackett G Montorsi P Montorsi F Vlachopoulos C Kloner R Sharlip I Miner M Erectile dysfunction and coronary artery disease prediction: evidence-based guidance and consensus Int J Clin Pract 2010 64 848 857 10.1111/j.1742-1241.2010.02410.x 20584218

[B10] Thompson I Tangen C Goodman P Probstfield J Moinpour C Coltman C Erectile dysfunction and subsequent cardiovascular disease JAMA 2005 294 2996 3002 10.1001/jama.294.23.2996 16414947

[B11] Inman B Sauver J Jacobson D McGree M Nehra A Lieber M Roger V Jacobsen S A population-based, longitudinal study of erectile dysfunction and future coronary artery disease Mayo Clin Proc 2009 84 108 113 10.4065/84.2.108 19181643 PMC2664580

[B12] Araujo A Hall S Ganz P Chiu G Rosen R Kupelian V Travison T McKinlay J Does erectile dysfunction contribute to cardiovascular disease risk prediction beyond the Framingham risk score J Am Coll Cardiol 2010 55 350 356 10.1016/j.jacc.2009.08.058 20117441 PMC2845313

[B13] Montorsi P Ravagnani PM Galli S Rotatori F Briganti A Salonia A Rigatti P Montorsi F The artery size hypothesis: a macrovascular link between erectile dysfunction and coronary artery disease Am J Cardiol 2005 96 Suppl 19M 23M 16387561 10.1016/j.amjcard.2005.07.006

[B14] Sisson SB Camhi SM Church TS Martin CK Tudor-Locke C Bouchard C Earnest CP Smith SR Newton RL Jr Rankinen T Katzmarzyk PT Leisure time sedentary behaviour, occupational/domestic physical activity and metabolic syndrome in U.S. men and women Metab Syndr Relat Disord 2009 7 529 536 10.1089/met.2009.0023 19900152 PMC2796695

[B15] Hu G Qiao Q Tuomilehto J Balkau B Borch-Johnsen K Pyorala K for the DECODE study group Prevalence of the metabolic syndrome and its relation to all-cause and cardiovascular mortality in nondiabetic European men and women Arch Intern Med 2004 164 1066 1067 10.1001/archinte.164.10.1066 15159263

[B16] Third report of the National Cholesterol Education Program (NCEP) expert panel on detection, evaluation and treatment of high blood cholesterol in adults (Adult Treatment Panel III) Final report Circulation 2002 106 3143 3421 12485966

[B17] Ainsworth B Haskell W Whitt M Irwin M Swartz A Strath S O'Brien W Basset D Schmitz K Emplaincourt P Jacobs D Leon A Compendium of physical activities: an update of activity codes and MET intensities Med Sci Sports Exerc 2000 32 498 516 10.1097/00005768-200009001-00009 10993420

[B18] Rosen R Riley A Wagner G Osterloh I Kirkpatrick J Mishra A The international index of erectile function (IIEF): a multidimensional scale for assessment of erectile dysfunction Urology 1997 6 822 830 10.1016/s0090-4295(97)00238-0 9187685

[B19] Prisant LM Pasi M Jupin D Prisant ME Assessment of repeatability and correlates of arterial compliance Blood Press Monit 2000 7 231 235 10.1097/00126097-200208000-00005 12198339

[B20] Resnick LM Militianu D Cunnings AJ Pipe JG Evelhoch JL Soulen RL Lester MA Pulse waveform analysis of arterial compliance: relation to other techniques, age, and metabolic variables Am J Hypertens 2000 13 1243 1249 10.1016/S0895-7061(00)01219-X 11130766

[B21] Wilson AM O'Neal D Nelson CL Prior DL Best JD Jenkins AJ Comparison of arterial assessments in low and high vascular disease groups Am J Hypertens 2004 17 285 291 10.1016/j.amjhyper.2003.10.009 15062880

[B22] Zimlichman R Shargorodsky M Boaz M Duprez D Rahn KH Rizzoni D Payeras AC Hamm C McVeigh G Determination of arterial compliance using blood pressure waveform analysis with the CR-2000 system: reliability, repeatability, and establishment of normal values for healthy European population - the seven European sites study (SESS) Am J Hypertens 2005 18 65 71 15691619 10.1016/j.amjhyper.2004.08.013

[B23] Bacon CG Mittleman MA Kawachi I Giovannucci E Glasser DB Rimm EB A prospective study of risk factors for erectile dysfunction J Urol 2006 176 217 221 10.1016/S0022-5347(06)00589-1 16753404

[B24] Kratzik CW Lackner JE Märk I Rücklinger E Schmidbauer J Lunglmayr G Schatzl G How much physical activity is needed to maintain erectile function? Results of The Androx Vienna Municipality study Eur Urol 2009 55 509 517 10.1016/j.eururo.2008.02.020 18359146

[B25] Esposito K Giugliano F Di Palo C Giugliano G Marfella R D'Andrea F D'Armiento M Giugliano D Effect of lifestyle changes on erectile dysfunction in obese men. A randomized controlled trial JAMA 2004 291 2978 2984 10.1001/jama.291.24.2978 15213209

[B26] Teles AG Carreira M Alarcão V Sociol D Aragüés JM Lopes L Mascarenhas M Costa JG Prevalence, severity, and risk factors for erectile dysfunction in a representative sample of 3,548 Portuguese men aged 40 to 69 years attending primary healthcare centers: results of the Portuguese erectile dysfunction study J Sex Med 2008 5 1317 1324 10.1111/j.1743-6109.2007.00745.x 18194181

[B27] Christensen BS Grønbaek M Pedersen BV Graugaard C Frisch M Horansanli K Boylu U Kendirci M Miroglu C Associations of unhealthy lifestyle factors with sexual inactivity and sexual dysfunctions in Denmark J Sex Med 2011 in press 10.1111/j.1743-6109.2011.02291.x 21569211

[B28] Hadaegh F Zabetian A Khalili D Safarkhani M James WP Azizi F A new approach to compare the predictive power of metabolic syndrome defined by a joint interim statement versus its components for incident cardiovascular disease in Middle East Caucasian residents in Tehran J Epidemiol Community Health 2010 in press 10.1136/jech.2010.117697 21051780

[B29] Bal K Öder M Şahin A Karataş C Demir Ö Can E Gümüş B Özer K Şahin O Esen A Prevalence of metabolic syndrome and its association with erectile dysfunction among urologic patients: metabolic backgrounds of erectile dysfunction Urology 2007 69 356 360 10.1016/j.urology.2006.09.057 17275074

[B30] Selvin E Burnett AL Platz EA Prevalence and risk factors for erectile dysfunction in the US Am J Med 2007 120 151 157 10.1016/j.amjmed.2006.06.010 17275456

[B31] Ko DT Hebert PR Coffey CS Sedrakyan A Curtis JP Krumholz HM Beta-blocker therapy and symptoms of depression, fatigue, and sexual dysfunction JAMA 2002 288 351 357 10.1001/jama.288.3.351 12117400

[B32] Düsing R Effect of the angiotensin II antagonist valsartan on sexual function in hypertensive men Blood Press Suppl 2003 2 29 34 14761074 10.1080/08038020310021967

[B33] Doğru MT Başar MM Simşek A Yuvanç E Güneri M Ebinç H Batislam E Effects of statin treatment on serum sex steroids levels and autonomic and erectile function Urology 2008 71 703 707 10.1016/j.urology.2007.11.059 18387399

[B34] Ford ES The metabolic syndrome and C-reactive protein, fibrinogen, and leukocyte count: findings from the Third National Health and Nutrition Examination Survey Atherosclerosis 2003 168 351 358 10.1016/S0021-9150(03)00134-5 12801619

[B35] Church TS Finley CE Earnest CP Kampert JB Gibbons LW Blair SN Relative associations of fitness and fatness to fibrinogen, white blood cell count, uric acid and metabolic syndrome Int J Obes 2002 26 805 813 10.1038/sj.ijo.0802001 12037651

[B36] Myint PK Luben RN Wareham NJ Welch AA Bingham SA Khaw KT Physical activity and fibrinogen concentrations in 23,201 men and women in the EPIC-Norfolk population-based study Atherosclerosis 2008 198 419 425 10.1016/j.atherosclerosis.2007.09.021 17977548

[B37] Sinha S Luben RN Welch A Bingham S Wareham NJ Day NE Khaw KT Fibrinogen and cigarette smoking in men and women in the European Prospective Investigation into Cancer in Norfolk (EPIC-Norfolk) population Eur J Cardiovasc Prev Rehabil 2005 12 144 150 15785300 10.1097/01.hjr.0000140719.09768.e2

[B38] Vlachopoulos C Aznaouridis K Ioakeimidis N Rokkas K Vasiliadou C Alexopoulos N Stefanadi E Askitis A Stefanadis C Unfavourable endothelial and inflammatory state in erectile dysfunction patients with or without coronary artery disease Eur Heart J 2006 27 2640 2648 10.1093/eurheartj/ehl341 17056702

[B39] Pohjantähti-Maaroos H Palomäki A Kankkunen P Laitinen R Husgafvel S Oksanen K Circulating oxidized low-density lipoproteins and arterial elasticity: comparison between men with metabolic syndrome and physically active counterparts Cardiovasc Diabetol 2010 9 41 10.1186/1475-2840-9-41 20727144 PMC2931500

[B40] Ge JY Li XL Zhang HF Xu Q Tong M Wang JG Elasticity indices of large and small arteries in relation to the metabolic syndrome in Chinese Am J Hypertens 2008 21 143 147 10.1038/ajh.2007.26 18188165

[B41] Kaya C Ergelen M Ilktac A Karaman I Impaired elasticity of aorta in patients with erectile dysfunction Urology 2007 70 558 562 10.1016/j.urology.2007.04.018 17688916

[B42] Vlachopoulos C Aznaouridis K Ioakeimidis N Rokkas K Tsekoura D Vasiliadou C Stefanadi E Askitis A Stefanadis C Arterial function and intima-media thickness in hypertensive patients with erectile dysfunction J Hypertens 2008 26 1829 1836 10.1097/HJH.0b013e3283050886 18698219

[B43] Pohjantähti-Maaroos H Palomäki A Comparison of metabolic syndrome subjects with and without erectile dysfunction - levels of circulating oxidised LDL and arterial elasticity Int J Clin Pract 2011 65 274 280 10.1111/j.1742-1241.2010.02595.x 21314864

[B44] Kaiser D Billups K Mason C Wetterling R Lundberg J Bank A Impaired brachial artery endothelium-dependent and -independent vasodilation in men with erectile dysfunction and no other clinical cardiovascular disease J Am Coll Cardiol 2004 43 179 184 10.1016/j.jacc.2003.07.042 14736434

[B45] Yavuzgil O Altay B Zoghi M Gürgün C Kayıkçıoğlu M Kültürsay H Endothelial function in patients with vasculogenic erectile dysfunction Int J Cardiol 2005 103 19 26 10.1016/j.ijcard.2004.07.004 16061118

[B46] Sugawara J Inoue H Haysashi K Yokoi T Kono I Effect of low-intensity aerobic exercise training on arterial compliance in postmenopausal women Hypertens Res 2004 27 897 901 10.1291/hypres.27.897 15894828

[B47] Elosua R Molina L Fito M Arquer A Sanchez-Quesada JL Covas MI Ordonez-Llanos J Marrugat J Response of oxidative stress biomarkers to a 16-week aerobic physical activity program, and to acute physical activity, in healthy young men and women Atherosclerosis 2003 167 327 334 10.1016/S0021-9150(03)00018-2 12818416

[B48] Miyachi M Kawano H Sugawara J Takahashi K Hayashi K Yamazaki K Tabata I Tanaka H Unfavorable effects of resistance training on central arterial compliance. A randomized intervention study Circulation 2004 110 2858 2863 10.1161/01.CIR.0000146380.08401.99 15492301

[B49] Muñoz MD Olcina G Timón R Robles MC Caballero MJ Maynar M Effect of different exercise intensities on oxidative stress markers and antioxidant response in trained cyclists J Sports Med Phys Fitness 2010 50 93 98 20308979

[B50] Mancia G Bousquet P Elghozi JL Esler M Grassi G Julius S Reid J Van Zwieten PA The sympathetic nervous system and the metabolic syndrome J Hypertens 2007 25 909 920 10.1097/HJH.0b013e328048d004 17414649

[B51] Arnold JM Fitchett DH Howlett JG Lonn EM Tardif JC Resting heart rate: a modifiable prognostic indicator of cardiovascular risk and outcomes? Can J Cardiol 2008 24 SupplA 3A 8A 18437251 10.1016/s0828-282x(08)71019-5 PMC2787005

[B52] Tomiyama H Hashimoto H Tanaka H Matsumoto C Odaira M Yamada J Yoshida M Shiina K Nagata M Yamashina A baPWV/cfPWV Collaboration Group Synergistic relationship between changes in the pulse wave velocity and changes in the heart rate in middle-aged Japanese adults: a prospective study J Hypertens 2010 28 687 694 10.1097/HJH.0b013e3283369fe8 20051904

[B53] Corona G Mannucci E Forti G Maggi M Hypogonadism, ED, metabolic syndrome and obesity: a pathological link supporting cardiovascular diseases Int J Androl 2009 32 587 598 10.1111/j.1365-2605.2008.00951.x 19226407

[B54] Revnic CR Nica AS Revnic F The impact of physical training on endocrine modulation, muscle physiology and sexual functions in elderly men Arch Gerontol Geriatr 2007 44 Suppl 1 339 342 17317472 10.1016/j.archger.2007.01.046

[B55] Vlachopoulos C Rokkas K Ioakeimidis N Stefanadis C Inflammation, metabolic syndrome, erectile dysfunction and coronary artery disease: common links Eur Urol 2007 52 1590 1600 10.1016/j.eururo.2007.08.004 17707576

[B56] Cappelleri J Rosen R Smith M Mishra A Osterloh I Diagnostic evaluation of the erectile function domain of the international index of erectile function Urology 1999 54 346 351 10.1016/S0090-4295(99)00099-0 10443736

[B57] O'Donnell AB Araujo AB Goldstein I McKinlay JB The validity of a single-question self-report of erectile dysfunction. Results from the Massachusetts Male Aging Study J Gen Intern Med 2005 20 515 519 10.1111/j.1525-1497.2005.0076.x 15987326 PMC1490137

